# Application and accuracy of artificial intelligence-derived large language models in patients with age related macular degeneration

**DOI:** 10.1186/s40942-023-00511-7

**Published:** 2023-11-18

**Authors:** Lorenzo Ferro Desideri, Janice Roth, Martin Zinkernagel, Rodrigo Anguita

**Affiliations:** 1 Department of Ophthalmology, Inselspital, University Hospital of Bern, Bern, Switzerland; 2 Bern Photographic Reading Center, Inselspital, Bern University Hospital, University of Bern, Bern, Switzerland; 3 Moorfields Eye Hospital NHS Foundation Trust, City Road, London, EC1V 2PD UK

**Keywords:** LLMs, Large language models, Artificial Intelligence, Artificial intelligence in ophthalmology, Macular edema, Wet macular degeneration, Dry macular degeneration

## Abstract

**Introduction:**

Age-related macular degeneration (AMD) affects millions of people globally, leading to a surge in online research of putative diagnoses, causing potential misinformation and anxiety in patients and their parents. This study explores the efficacy of artificial intelligence-derived large language models (LLMs) like in addressing AMD patients' questions.

**Methods:**

ChatGPT 3.5 (2023), Bing AI (2023), and Google Bard (2023) were adopted as LLMs. Patients’ questions were subdivided in two question categories, (a) general medical advice and (b) pre- and post-intravitreal injection advice and classified as (1) accurate and sufficient (2) partially accurate but sufficient and (3) inaccurate and not sufficient. Non-parametric test has been done to compare the means between the 3 LLMs scores and also an analysis of variance and reliability tests were performed among the 3 groups.

**Results:**

In category a) of questions, the average score was 1.20 (± 0.41) with ChatGPT 3.5, 1.60 (± 0.63) with Bing AI and 1.60 (± 0.73) with Google Bard, showing no significant differences among the 3 groups (p = 0.129). The average score in category b was 1.07 (± 0.27) with ChatGPT 3.5, 1.69 (± 0.63) with Bing AI and 1.38 (± 0.63) with Google Bard, showing a significant difference among the 3 groups (p = 0.0042). Reliability statistics showed Chronbach’s α of 0.237 (range 0.448, 0.096–0.544).

**Conclusion:**

ChatGPT 3.5 consistently offered the most accurate and satisfactory responses, particularly with technical queries. While LLMs displayed promise in providing precise information about AMD; however, further improvements are needed especially in more technical questions.

## Introduction

Age-related macular degeneration (AMD) represents a leading cause of visual loss affecting around 200 million people worldwide and its prevalence is steadily increasing [1]. In 2040 AMD prevalence is expected to raise up to 288 million people worldwide [2].

Given this alarming epidemiological data, AMD represent an important social and economic burden; nonetheless, a growing trend of AMD patients seeking diagnosis online is expected and this scenario poses a multifaceted challenge [3]. This represents a social issue as it can lead to misinformation and unnecessary anxiety for patients. In fact, many patients affected with AMD often seek online answers about their disease, the possible treatment options, and their visual prognosis, but often the information reported can be wrong, inaccurate, and sometimes misleading [4]. Addressing this issue requires promoting digital health literacy, offering reliable online resources, and educating patients on the significance of consulting healthcare professionals for accurate diagnosis and proper care [5, 6]. An integrated approach is essential to harness the benefits of digitalization while mitigating its challenges in healthcare [7].

In recent years, there has been a significant increase in the use of artificial intelligence (AI) in healthcare sector and in ophthalmological field [8]. This growth is due in part to the advancements in AI subfields such as data visualization, speech recognition, and natural language processing, which facilitates patients to access clinical information through large language models (LLMs) [9]. LLMs are AI- derived models trained extensively on text data using deep learning (DL) techniques and they are capable to understand and replicate human-like responses by analyzing patterns and context in their training data. LLMs are adept at generating relevant responses to a wide range of prompts or questions [10].

Recent studies have investigated the role of LLMs in generating reliable information for the patients with several ophthalmological diseases, including uveitis, ocular tumors, glaucoma, and others [11–14]. A recent study showed the potential of ChatGPT 3.5 in creating ophthalmic discharge summaries and operative notes, concluding that an adequate training of LLMs on these task with human verification may have a positive impact on healthcare [15].

In this study, we tasked with responding 3 of the most common LLMs with the most frequent questions of patients with AMD. The aim of this study is to assess the accuracy and feasibility of LLMs in addressing patients with AMD and helping them to acquire more validated information about their health status condition, prognosis, and doubts regarding their available treatment options.

## Methods

In our investigation into the quality and reliability of information provided by LLMs. In this study the authors selected three of the most widely used and freely available LLMs, all of which were posed with the most common questions formulated by patients suffering from AMD. The LLMs under scrutiny were ChatGPT 3.5 (2023) by OpenAI, Bing AI (2023) powered by GPT-4 (2023) and developed by Microsoft, and Google Bard by Google. To systematically assess their performance, we elaborated a set of questions, dividing them into two distinct categories: 15 questions related to medical advice and the most common questions of patients, as outlined in Table 1, and 13 technical questions regarding pre- and post-intravitreal injections advice, detailed in Table 2.

**Table 1:** Medical advice general questions in patients with macular degeneration

Questions	ChatGPT	Bing AI	Google Bard
1. How common is AMD?	1	1	1
2. In a patient with established diagnosis of AMD, what is the chance the other eye is affected with AMD?	2	1	3
3. What is the underlying cause of AMD?	1	2	2
4. Is AMD inherited?	1	1	1
5. What is dry AMD?	1	2	1
6. What is wet AMD?	1	1	1
7. What are the chances it converts into wet AMD?	2	1	2
8. What is the best treatment for dry and wet AMD?	2	2	3
9. How can I know that my dry AMD converted into wet AMD? What are the symptoms?	1	2	1
10. How can I test myself for AMD? How often should I perform Amsler grid examination?	1	1	2
11. I have been diagnosed with AMD. Are there any eyeglasses or contact lenses I can wear to improve my condition?	1	2	2
12. How can I slow down AMD progression naturally?	1	3	1
13. Will I lose vision/go blind?	1	1	1
14. Do vitamins and oral nutritional supplements help for AMD?	1	2	1
15. What happens if AMD is left untreated?	1	2	2
16. Can I drive with AMD?	1	1	1

	Tests of Normality					
	Kolmogorov-Smirnov^a^			Sharpiro-Wilk		
	Statistic	df	Sig	Statistic	df	Sig
ChatGPT	,492	16	,000	,484	16	,000
BingAl	,314	16	,000	,750	16	,001
GoogleBard	,343	16	,000	,738	16	,000

^a^Lilliefors Significance Correction

1 = Accurate and sufficient

2 = Partially accurate and sufficient

3 = Inaccurate and insufficient

**Table 2:** Pre- and post-intravitreal injections advice questions in patients with macular degeneration

**Questions**	ChatGPT	Bing AI	Google Bard
1. What is it an intravitreal injection?	1	2	1
2. What are the risks associated with intravitreal injections?	1	2	1
3. How do these anti-VEGF agents work? Do they treat only the wet AMD form?	1	2	2
4. Are there any medications against the dry form? How do they work?	2	1	3
5. I have problems to come every month to the hospital for AMD intravitreal injections. Are there any drugs allowing me a more extended treatment interval?	1	2	3
7. Should I take any medicaments after the intravitreal injection? For how long?	1	2	1
8. What should avoid doing after intravitreal injection?	1	2	1
9. Can I exercise and/or lift objects after intravitreal injection? And can I go swimming?	1	2	1
10. Can I wear my contact lenses after anti-VEGF injection?	1	3	1
11. I see a mobile bubble moving in the visual field since I have been injected. Should I worry about that?	1	2	1
12. After the anti-VEGF intravitreal injection, a large blood effusion has appeared in my conjunctiva. This blood effusion is really scaring me. What can it be? What should I do?	1	1	1
13. My eye keeps on tearing after the anti-VEGF intravitreal injection and it still seems to be reddened. What can it be? What should I do?	1	1	1
14. I have been injected some days ago with an anti-VEGF intravitreal injection. Now I feel severe pain in my eye, which is reddened, and I noticed a severe visual impairment. What can it be? What should I do?	1	1	1

1 = Accurate and sufficient

2 = Partially accurate and sufficient

3 = Inaccurate and insufficient

The responses generated by these LLMs were discussed and evaluated after common agreement by three experienced retina specialists (with at least 8 years of clinical experience). Their evaluations led to categorizations based on accuracy and sufficiency. Responses were classified as 'Accurate and Sufficient' if they were both correct and comprehensive. 'Partially Accurate and Sufficient' was assigned when responses contained minor inaccuracies but still provided substantial and understandable information. Lastly, 'Inaccurate' denoted answers that were entirely incorrect or contained critical errors rendering them unreliable.

Statistical analysis was conducted by using the SPSS program (IBM SPSS Statistics, version 25). Descriptive analysis (including frequency, means and standard deviation) and normality distribution test (Shapiro–Wilk) have been done. A non-parametric Kruskal–Wallis test has been subsequently performed, given the abnormal distribution of the data, to compare average scores across the three LLMs. Reliability test was also performed by measuring Cronbach α coefficient. A p-value of less than 0.05 was considered statistically significant.

## Results

**Fig. 1:**
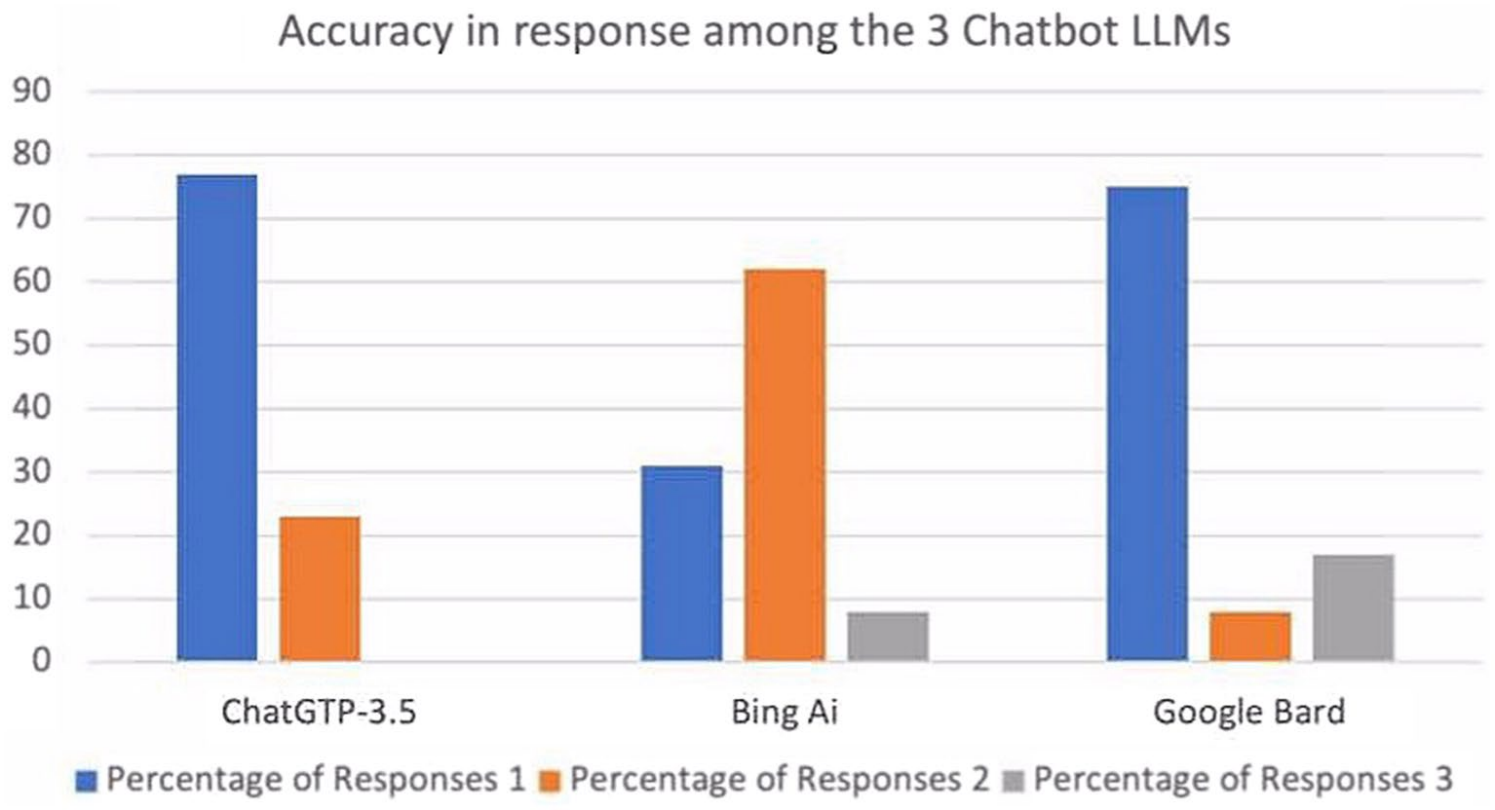
Accuracy of response among the 3 Chatbot large language models in patients with macular degeneration

In the group of medical advice general questions, ChatGPT 3.5 showed that 80.0% (n = 12) of the response were classified as accurate and sufficient and the remaining 20% (n = 3) as partially accurate and sufficient. Bing AI reported 46.7% (n = 7) of the response classified as accurate and sufficient and another 46.7% classified as partially accurate and sufficient, while only 6.7% (n = 1) were reputed inaccurate and insufficient. Google Bard was referred with 53.3% (n = 8) of the answers accurate and sufficient, 33.3% (n = 5) as partially accurate and sufficient and the remaining 13.3% (n = 2) were inaccurate and insufficient **(**Fig. 1**)**. In this first group of question, the average score was 1.20 (± 0.41) with ChatGPT 3.5, 1.60 (± 0.63) with Bing AI and 1.60 (± 0.73) with Google Bard, showing no significant differences among the 3 groups (p = 0.129).

In the second group of questions (pre- and post-intravitreal injections advice questions), ChatGPT 3.5 answered 76.9% (n = 10) of the questions accurately and sufficiently and 23.1% (n = 3) partially accurately and sufficiently. Differently, Bing AI showed 30.8% (n = 4) of the response as accurate and sufficient, 61.5% (n = 8) of them as partially accurate and sufficient and the remaining 7.7% (n = 1) of them as inaccurate and insufficient. Google Bard answered accurately and sufficiently in 75.0% (n = 9) of the questions, partially accurately and sufficiently in 8.3% (n = 1) of them and inaccurately and insufficiently in the remaining 26.7% (n = 2). The average score was 1.07 (± 0.27) with ChatGPT 3.5, 1.69 (± 0.63) with Bing AI and 1.38 (± 0.63) with Google Bard, showing a significant difference among the 3 groups (p = 0.0042).

Reliability statistics showed Chronbach’s α of 0.237 (range 0.448, 0.096–0.544), indicating an overall low agreement between the 3 LLMs.

## Discussion

Our research offers a comprehensive assessment of ChatGPT 3.5, Bing AI, and Google Bard in their ability to respond effectively to commonly asked questions about AMD from patients or their parents. To improve the integrity of our evaluation, the Chatbot LLMs-generated responses were thoroughly reviewed by 3 distinct experienced retina specialists. Our results showed that on average these 3 LLMs have the potential to provide accurate answers to AMD-related queries; however, the relatively low results in reliability test showed a relatively low level of agreement between the 3 LLMs. Our results emphasize that ChatGPT 3.5 consistently performed well in providing accurate and sufficient information, particularly excelling in technical questions related to pre- and post-intravitreal injections. Nonetheless, no response from ChatGPT 3.5 were characterized as inaccurate and insufficient. Differently, Bing AI displayed mixed performance, while Google Bard showed strength in certain aspects but also exhibited some inaccuracies. Although ChatGPT 3.5 has outperformed the other 2 LLMs in terms of accuracy and reliability of the answers, our findings suggest that LLMs still give different levels of performance and they cannot still be considered interchangeable tools in the providing accurate information for patients with AMD.

To the best of our knowledge, this the first study to investigate the utility of LLMs focusing specifically on addressing patients with AMD with general questions on technical questions on pre-and post-operative management. We found that LLMs may provide a promising supportive role to patients, which may be sometimes lost and confused about their condition, its management, treatment options and prognosis. It has been widely reported that patient’s satisfaction is highly dependent on an appropriate information regarding their condition [16]; however, previous studies have reported that the online information about ophthalmological conditions may be often inaccurate and misleading [17, 18]. Nowadays, we are presented with significant worldwide challenges and prospects as a result of several factors: the global population is growing with a shift to an aging demographic, diagnostic capabilities are improving, and treatment options are expanding [19]. Considering the increasing requests, ophthalmologists may not always be readily accessible, in contrast with internet and Chatbot LLMs platforms, which are already widely used by the global community [20].

ChatGPT 3.5, BingAI and Bard, accounting as 3 of the most prominent LLMs are AI-based services that can be easily accessed via internet. These LLMs have been developed in a way allowing to understand and respond to user questions and instructions. Furthermore, they have been extensively trained on diverse text sources, including articles, books, and websites, enabling them to generate responses that mimic human language when prompted [21].

In this scenario, LLMs offer the advantage of accessibility, allowing patients to quickly access information and obtain answers at their convenience, a particularly significant advantage in remote or isolated areas, and in some cases translating medical information into patients' native languages [10, 15, 22]. Additionally, responses generated by LLMs are more comprehensible than medical jargon, further enhancing their utility [23].

A previous study evaluated the general responses generated by ChatGPT 3.5 regarding different retinal diseases, including AMD, central serous chorioretinopathy and retinal vein occlusions. They rated 45% of the LLM-generated answers as very good, 26% as minor non-harmful inaccurate and only 17% as markedly misinterpretable [3]. In another study published by Anguita et *al.*, LLMs were shown to potentially play a beneficial role in vitreoretinal care, also if proper patient education on their use is still needed [12].

Another study evaluated the accuracy of GTP at diagnosing glaucoma based on specific clinical case descriptions with comparison to the performance of senior ophthalmology resident trainees. In this study, ChatGPT 3.5 demonstrated a diagnostic accuracy of 72.7% when diagnosing primary and secondary glaucoma cases, outperforming some senior ophthalmology residents who achieved an accuracy of 54.5% to 72.7%. These findings suggested that ChatGPT 3.5 has the potential to assist in clinical settings for efficient and objective glaucoma diagnoses, particularly in primary care offices and eye care practices [13].

Another study evaluated the capacity of ChatGPT 3.5 to improve the readability of patient-targeted health information on uveitis. ChatGPT 3.5 generated responses with significantly lower Flesch Kincaid Grade Level scores and fewer complex words when asked to simplify the language, making the content more accessible to the average American reader. The findings suggested that ChatGPT 3.5 has the potential to assist healthcare professionals in creating more understandable uveitis information for patients and enhancing the overall accessibility of healthcare content [11].

Furthermore, it might be important to question that readability and simplifying language might come at the cost of accuracy of information. It should be further investigated if ChatGPT3.5 and the others 2 LLMs can correctly decide which part of the information should be omitted and accurately translate medical knowledge to simple terms without compromising the facts.

Nonetheless, some limitations are present in the study including the relative low sample of tasks for LLMs and the adoption of only 3 LLMs. Further studies should investigate the applicability of other advanced LLMs, including ChatGPT 4.0, with a larger sample of tasks in patients with AMD.

In a healthcare landscape where accessibility and patient education are crucial, LLMs offer a valuable tool, bridging communication gaps and providing understandable medical information. This study contributes to the growing body of evidence highlighting LLMs' utility in healthcare, particularly in addressing specific patient queries within the context of AMD.

## Conclusion

The future integration of Chatbots LLMs into the ophthalmologists’ daily clinical practice may represent a priceless opportunity for both eye specialists and patients with AMD. Our study showed that ChatGPT 3.5 consistently offered the most accurate responses, particularly with technical queries. Overall the 3 LLMs displayed promise in providing precise information about AMD; however, further improvements are warranted especially in more technical questions. Future, larger-scale, and real-life studies, possibly adopting questionnaire directly interrogating patients’ satisfaction and feasibility to adopt LLMs in their everyday life, may address us on the reach of these novel AI-tools to improve patients and physicians’ life.

## Data Availability

All data are available and kept in Inselspital protected database and.
